# Extracorporeal membrane oxygenation for refractory acute respiratory distress syndrome in severe malaria

**DOI:** 10.1186/1475-2875-12-306

**Published:** 2013-08-31

**Authors:** Carlos Alves, Jen-Ting Chen, Nina Patel, Darryl Abrams, Paulo Figueiredo, Lurdes Santos, António Sarmento, José Artur Paiva, Matthew Bacchetta, May-Lin Wilgus, Roberto Roncon-Albuquerque, Daniel Brodie

**Affiliations:** 1 Department of Infectious Diseases, Centro Hospitalar S. João, Porto, Portugal; 2 Division of Cardiothoracic Surgery, Columbia University College of Physicians and Surgeons, 630 W. 168th St., PH 8 East, New York, NY 10032, USA; 3 Department of Intensive Care Medicine, Centro Hospitalar S.João, Porto, Portugal

**Keywords:** Extracorporeal membrane oxygenation, Acute respiratory distress syndrome, Malaria, *Plasmodium falciparum*

## Abstract

**Background:**

Severe malaria may be complicated by the acute respiratory distress syndrome (ARDS), which is associated with a high mortality. In the present report, a series of three cases of imported malaria complicated by refractory severe ARDS supported with extracorporeal membrane oxygenation (ECMO) is presented.

**Methods:**

One female and two male adult patients (ages 39 to 53) were included. Two patients had *Plasmodium falciparum* infection and one patient had *Plasmodium vivax* and *Plasmodium ovale* co-infection. Anti-malarial therapy consisted in intravenous quinine (in two patients) and intravenous quinidine (in one patient), plus clindamycin or doxycycline.

**Results:**

Despite lung protective ventilation, a conservative strategy of fluid management, corticosteroids (two patients), prone position (two patients) and inhaled nitric oxide (one patient), refractory severe ARDS supervened (PaO_2_ to FiO_2_ ratio 68) and venovenous ECMO was then initiated. In one patient, a bicaval dual-lumen cannula was inserted; in the two other patients, a two-site configuration was used. Two patients survived to hospital-discharge (duration of ECMO support: 8.5 days) and one patient died from nosocomial sepsis and multi-organ failure after 40 days of ECMO support.

**Conclusions:**

ECMO support allowed adequate oxygenation and correction of hypercapnia under lung protective ventilation, therefore reducing ventilator-induced lung injury. ECMO referral should be considered early in malaria complicated by severe ARDS refractory to conventional treatment.

## Background

Malaria is responsible for more than one million deaths annually, the majority of which occur in sub-Saharan Africa, with *Plasmodium falciparum* as the predominant organism [1]. In non-endemic regions, most cases occur in non-immune or semi-immune travellers returning from endemic countries who do not adhere to malaria chemoprophylaxis [2,3]. Such cases may be particularly severe, with in-hospital mortality rates reported as 11 to 40% [2,4-6]. Acute respiratory distress syndrome (ARDS), which may develop in either the acute or late phases of infection, has been reported in 5 to 25% of adults with severe *P. falciparum* and in 1 to 10% of patients with severe *Plasmodium vivax*[1], with an attributable mortality of 20% in developed countries [5].

Extracorporeal membrane oxygenation (ECMO), referring to an extracorporeal circuit that directly oxygenates and removes carbon dioxide from the blood, may be considered in patients with ARDS whose pulmonary injury is so severe that positive-pressure ventilation alone is insufficient to maintain adequate gas exchange, or when adherence to lung-protective ventilation strategies results in unacceptable levels of hypercapnia and acidaemia [7]. In most cases of ECMO for severe ARDS, venovenous ECMO is utilized, in which blood is withdrawn from and returned to a central vein. Recently, there has been increasing interest in ECMO as a result of advances in extracorporeal technology, with more efficient oxygenators and lower rates of complications, along with several reports of improved survival with ECMO for severe ARDS [8-11]. However, reports of ECMO for severe ARDS in patients with malaria are uncommon, with only two cases reported separately in the literature [5,12], as well as one case of extracorporeal carbon dioxide elimination [13]. In the present report, a series of three cases of malaria complicated by severe ARDS refractory to conventional treatment and supported with ECMO is presented.

## Methods

### Case 1

**Figure 1:**
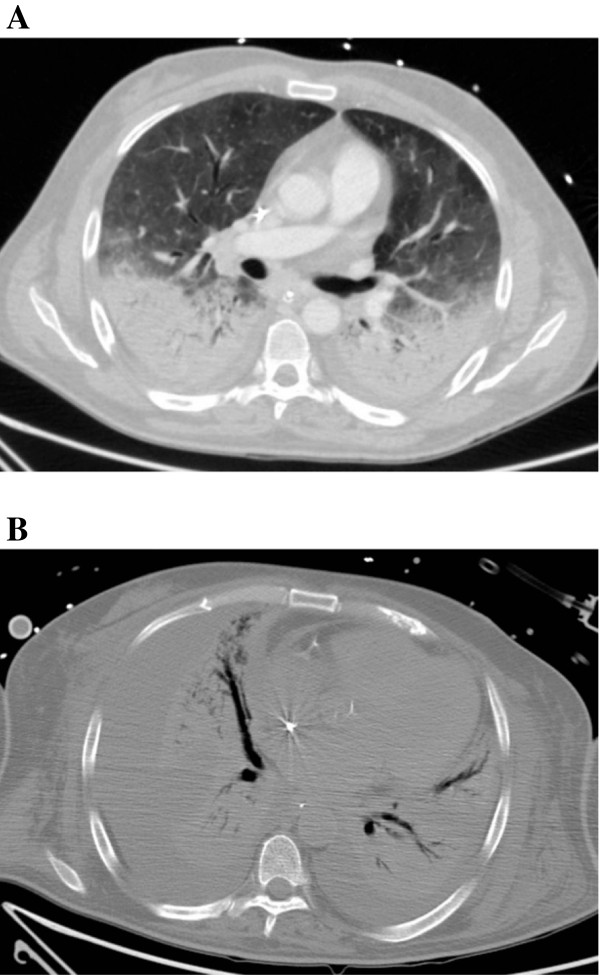
**Thoracic computed tomography (CT) before ECMO implantation (A) and after 40 days of ECMO support (B) in Patient 1. A**. Bilateral parenchymal consolidation in gravity-dependent areas and ground-glass-appearing opacities of lung parenchyma, compatible with severe ARDS. **B**. Extensive parenchymal consolidation and bilateral pleural effusions with associated compression atelectasis after 40 days of VV-ECMO.

A 53-year-old Portuguese man with a history of diabetes and myocardial infarction presented with five days of fever, chills, headache and myalgias (Table 1). The patient had been living in Angola, and he was not taking any malaria prophylaxis. The patient was evaluated at Hospital S João (Porto, Portugal), where vital signs and physical examination were unremarkable. Blood smear revealed *P. falciparum* (2% parasitaemia) and severe thrombocytopaenia (17,000 platelets/μl). The patient was initially admitted in a regular ward and oral quinine sulphate plus doxycycline were administered. However, shortly after admission, he developed vomiting with intolerance to oral drugs. In that context, intravenous quinine and doxycycline were started. No arrhythmia occurred during quinine treatment. The patient developed progressive hypoxemic respiratory failure requiring invasive mechanical ventilation (IMV) on hospital day 3 despite antimicrobials, diuresis, and clearance of his parasitaemia (Table 2). Blood cultures (obtained on hospital and ICU admission), urine culture and pneumococcal and Legionella urinary antigen tests, bacteriologic and virologic exams of tracheal secretions and bronchoalveolar lavage (performed immediately after tracheal intubation) were all negative. His thoracic CT scan revealed bilateral parenchymal consolidation in gravity-dependent areas and ground-glass-appearing opacities of lung parenchyma, compatible with severe ARDS (Figure 1A). Echocardiography was performed showing preserved ejection fraction and normal diastolic function. Moreover, plasma BNP was not elevated (98 pg/ml). Haemodynamic monitoring using the transpulmonary thermodilution technique and arterial pulse contour analysis (PiCCO^®^; Philips) was also performed before ECMO treatment, the results being consistent with ARDS (increased (23.7; normal range 3.0-7.0 ml/kg) extravascular lung water index (ELWI) and increased (6.6; normal range 1.0-3.0) pulmonary vascular permeability index (PVPI)), without intravascular volume overload (decreased (672; normal range 850–1000 ml/m^2^) intrathoracic blood volume index (ITBI) and decreased (538; normal range 680–800 ml/m^2^) global enddiastolic volume index (GEDI)). Cardiac index (3.6; normal range 3.0 - 5.0 l/min/m^2^) and systemic vascular resistance index (1838; normal range 1700–2400 dyn*s*cm^-5^*m^2^) were also recorded. On day 7 of IMV, his PaO_2_ was 69 mmHg with a FiO_2_ of 1.0 and PEEP of 13 cm of water, without improvement with a conservative strategy of fluid management, prone positioning or corticosteroids. ECMO was initiated with placement of a 21Fr right femoral venous drainage cannula and a 15Fr right internal jugular venous re-infusion cannula. The ventilator was then set to a lung-protective strategy (Table 2). On hospital day 20, ventilator-associated pneumonia occurred and *Pseudomonas aeruginosa* was isolated in cultures from bronchoalveolar lavage and blood. On hospital day 40 a new episode of VAP occurred and *Escherichia coli* was isolated in cultures from tracheal aspirate and on hospital day 48 septic shock with multi-organ failure supervened and *Pseudomonas aeruginosa* was isolated from the blood and tracheal aspirate. The patient died on hospital day 49 (ECMO day 40; Figure 1B). The only ECMO-related complication was thrombus formation in the oxygenator, requiring exchange on ECMO day 29.

**Table 1:** Characteristics of patients with malaria-related severe ARDS requiring ECMO

**Patient**	**1**	**2**	**3**
**Age (years)**	**53**	**39**	**46**
**Gender**	**Male**	**Male**	**Female**
**Country of origin**	**Angola**	**Mozambique**	**Uganda**
**Plasmodium species**	***P. falciparum***	***P. falciparum***	***P. vivax and P. ovale***
**Clinical deterioration (days)**			
Symptoms to hospital admission	5	3	14
Hospital to ICU admission	2	3	4
ICU admission to ECMO	9	14	1
IMV to ECMO	8	11	1
**Murray score**	**3.75**	**3.75**	**4.0**
**Before ECMO**			
Prone position	Yes	Yes	No
Nitric oxide	No	No	Yes
Renal replacement therapy	No	No	No
**During ECMO**			
Prone position	No	No	No
Nitric oxide	No	No	No
Renal replacement therapy	Yes	No	No
APACHE II *	**11**	**14**	**17**
**Mechanical ventilation (days)**	**48**	**25**	**17**
**ECMO (days)**	**40**	**7**	**10**
**ICU LOS (days)**	**49**	**46**	**23**
**ICU survival**	**NSurv**	**Surv**	**Surv**
**Hospital survival**	**NSurv**	**Surv**	**Surv**

APACHE II, Acute Physiology and Chronic Health Evaluation II; LOS, length of stay; ECMO, extracorporeal membrane oxygenation; IMV, invasive mechanical ventilation; NSurv, non-survivor; Surv, survivor. * First day of ICU admission.

**Table 2:** Respiratory and haematologic parameters before, during, and after ECMO

**Patient**	**1**	**2**	**3**
**ICU admission**			
Ventilation	SB	NIV	IMV
FiO_2_ (%)	85	50	100
pH / PaCO_2_ (mmHg)	7.50 / 36.2	7.41 / 39.4	7.41/39
Haemoglobin (g/dL)	11.4	12.0	9.6
Parasitaemia (%)	Negative	Negative	0.015
**Before ECMO**			
PC-peak / VC-plat (cmH_2_O)	32	30	34
Tidal volume (ml)	420	450	350
PEEP (cmH_2_O)	13	12	15
PaO_2_ / FiO_2_ (mmHg)	69	62	69
FiO_2_ (%)	100	100	100
pH / PaCO_2_ (mmHg)	7.36 / 72.9	7.35 / 73.1	7.41 / 39.0
Fluid balance (L/24h)	+0.35	+0.75	+0.52
**Day 2 on ECMO**			
PC-peak / VC-plat (cmH_2_O)	28	26	26
Tidal volume (ml)	280	340	210
PEEP (cmH_2_O)	10	10	10
FiO_2_ (%)	60	55	40
ECMO-BF (lpm)	3.0	3.0	3.5
ECMO-sweep (lpm)	2.0	3.0	3.0
pH / PaCO_2_ (mmHg)	7.40 / 47.0	7.44 / 54.0	7.45 / 36.0
Fluid balance (L/24h)	+1.55	+0.85	+2.70
**End of ECMO**			
PC-peak / VC-plat (cmH_2_O)	31	28	23
Tidal volume (ml)	110	560	385
PEEP (cmH_2_O)	8	8	12
FiO_2_ (%)	60	50	50
ECMO-BF (lpm)	3.0	3.0	3.3
ECMO-sweep (lpm)	7.0	2.5	1.5
pH / PaCO_2_ (mmHg)	7.22 / 33.0	7.43 / 38.5	7.43/42
Fluid balance (L/24h)	+1.85	+0.80	+0.48
**ICU discharge**			
Ventilation	NA	SB	SB
FiO_2_ (%)	NA	27	40
pH / PaCO_2_ (mmHg)	NA	7.47 / 30.7	7.44 / 38.0

ECMO-BF, blood flow through the ECMO circuit; ECMO-Sweep, sweep gas flow through the ECMO circuit; IVM, invasive mechanical ventilation; NA, not applicable; NIV, non-invasive ventilation; PaCO_2_, arterial carbon dioxide partial pressure; PEEP, positive end-expiratory pressure; PC-peak, peak inspiratory pressure under pressure control ventilation; SB, spontaneous breathing; VC-plat, plateau pressure under volume control ventilation.

### Case 2

**Figure 2:**
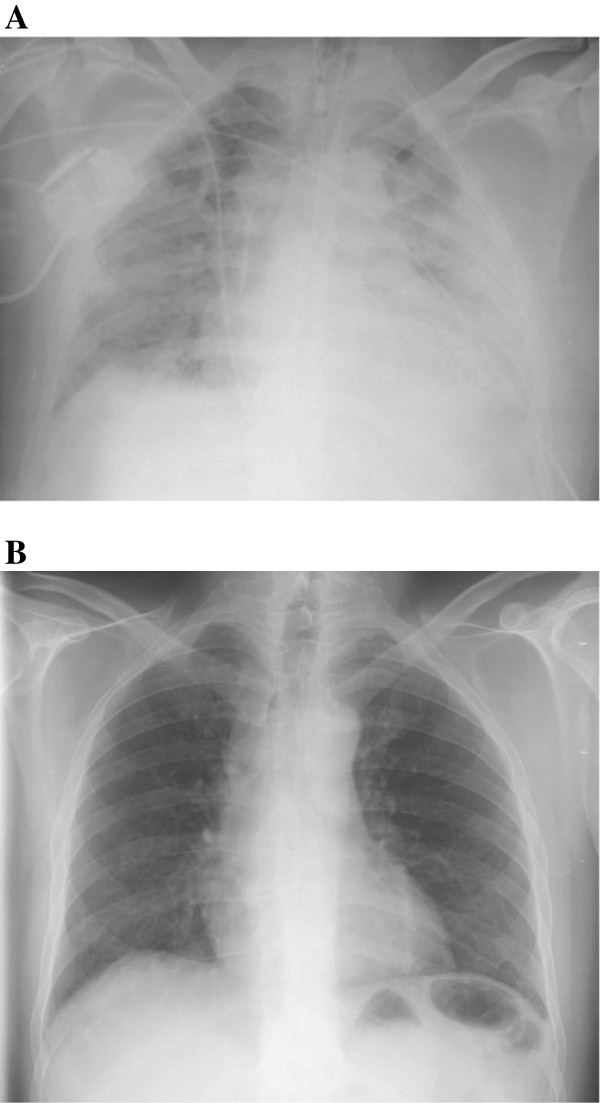
**Chest X-ray before ECMO implantation (A) and before hospital discharge (B) in Patient 2. A**. Bilateral parenchymal consolidation with air bronchograms, compatible with severe ARDS. **B**. Complete resolution of bilateral infiltrates.

A 39-year-old Portuguese man with a history of hypertension and a prior episode of malaria treated as an outpatient, presented with three days of fever, myalgias, headache, nausea and vomiting (Table 1). The patient had worked for several years in Mozambique, and he was not taking any malaria prophylaxis. On presentation, he was febrile and somnolent. Chest X-ray was normal. Blood smear revealed *P. falciparum* (3% parasitaemia) and thrombocytopaenia (80,000 platelets/μl). Taking into account the presence of vomiting with intolerance to oral drugs, the patient was treated with intravenous quinine and clindamycin. Despite negative cultures and clearance of his parasitaemia, hypoxemic respiratory failure developed on hospital day 6, requiring IMV (Table 2). Blood cultures (obtained on hospital and ICU admission), urine culture and urinary antigens (pneumococcal and Legionella), bacteriologic and virologic examinations of tracheal secretions and bronchoalveolar lavage (performed immediately after tracheal intubation) were all negative. Chest X-ray showed bilateral infiltrates and echocardiogram was unremarkable (Figure 2A). On day 11 of IMV, his PaO_2_ was 62 mmHg despite an FIO_2_ of 1.0, PEEP of 12 cm of water, a conservative strategy of fluid management, prone positioning, and corticosteroids. ECMO was then initiated with a 23Fr drainage cannula in the right femoral vein and 17Fr re-infusion cannula in the right internal jugular vein. The patient was successfully decannulated after seven days of ECMO support and weaned off IMV within the next seven days (Figure 2B). There were no ECMO-related complications. He was discharged home two weeks post-ICU discharge, where he has been living independently.

### Case 3

A 46-year-old American woman presented with two weeks of cyclic fevers, night sweats, and myalgias (Table 1). She had recently returned from Uganda, where she worked in a rural area and was adherent to atovaquone and proguanil malaria prophylaxis. She was treated empirically for chloroquine-resistant *P. falciparum* with oral quinine and doxycycline, however she did not tolerate oral therapy due to nausea and vomiting, and required admission to a local hospital for initiation of intravenous quinidine and clindamycin. Her vital signs, physical exam, and chest X-ray were unremarkable. Initial blood smears showed 0.5% parasitaemia. Once PCR testing confirmed *P. vivax* and *P. ovale* and excluded *P. falciparum*, her treatment was changed to chloroquine; however severe hypoxemic respiratory failure with bilateral infiltrates developed by hospital day 4, requiring IMV, at which point intravenous therapy was resumed. PaO_2_ was 68 mmHg despite an FIO_2_ of 1.0, PEEP of 15 cm of water, a conservative strategy of fluid management and inhaled nitric oxide (iNO) (Table 2). Echocardiogram demonstrated normal cardiac function. She was transferred to the Columbia University College of Physicians and Surgeons (New York, NY, USA), where she was placed on ECMO with a 27Fr bicaval dual-lumen cannula via the right internal jugular vein under fluoroscopic and echocardiographic guidance [14,15]. Cultures and blood smears in the ICU were negative for organisms. Anti-malarial therapy with intravenous quinidine and doxycycline was continued and she was successfully decannulated on ECMO day 10, and liberated from IMV seven days later. No arrhythmias occurred during quinine treatment. One month post-discharge, she was functioning independently. Two ECMO-related complications occurred: i) migration of the distal end of the cannula into a hepatic vein, identified by transesophageal echocardiogram on ECMO day 2 in the setting of a decrease in blood flow rates, followed by successful repositioning at the bedside; and ii) bleeding from the cannulation site and epistaxis that required a total of 8 units of packed red blood cells in the setting of low-level anticoagulation to maintain ECMO circuit integrity (mean activated partial thromboplastin time 46.5 seconds).

### Consent

Informed consent was obtained from the patients for publication of this case series and any accompanying images after explanation of the report objectives.

## Discussion

In this report, three cases of imported malaria complicated by severe ARDS supported with ECMO are presented. Severe ARDS may be a complication of *P. falciparum*, *P. vivax* or *P. ovale* infections. In accordance with the WHO guidelines the presence of ARDS is a defining criterion for severe malaria [16]. In all three cases, oxygenation and CO_2_ clearance through the membrane ECMO lung allowed the compliance to protective ventilation of the native lung with low tidal volumes (<6 ml/kg IBW) and low-fractional oxygen concentrations, limiting ventilator-induced lung injury [17]. However, permissive hypercapnia is frequently necessary to allow low tidal volume protective ventilation in severe ARDS. This could be potentially harmful in patients with severe malaria, given that cerebral oedema with elevated intracranial pressures can complicate cerebral malaria [18] and that hypercapnia causes cerebral vasodilatation and intracranial pressure elevation. Moreover, microvascular congestion and sequestration of parasitized red blood cells was shown to be a common feature in cerebral malaria, leading to impaired tissue perfusion that may cause diffuse cerebral ischemia and increased intracranial blood volume [19]. The use of ECMO in patients with severe malaria, allowing strict control of patient’s PaCO2, potentially limits intracranial pressure elevation.

Fluid management is crucial in patients with severe malaria. Taking into account that in these patients pulmonary edema secondary to increased pulmonary vascular permeability has been shown to be frequent, unpredictable and exacerbated by fluid loading, a conservative strategy of fluid management was followed in all three patients before and after ECMO initiation [20]. Moreover, a liberal strategy of fluid management in acute lung injury has been previously associated with prolonged duration of mechanical ventilation and intensive care [21].

ECMO is not without risks and there are no definitive criteria for ECMO treatment in adult ARDS. Therefore, ECMO should only be considered in adult patients with malaria and severe ARDS refractory to conventional treatment. The recently conducted CESAR trial showed favourable results of ECMO referral for adult patients with severe ARDS [8]. In that context, the same inclusion (Murray score >3.0 or pH <7.20) and exclusion (high pressure (>30 cm H_2_O of peak inspiratory pressure) or high FiO_2_ (>0.8) ventilation for more than seven days; intracranial bleeding; any other contraindication to limited heparinization) criteria for ECMO consideration in malaria and severe ARDS were used in the present case series.

Severe imported malaria is an important problem in the western world due to the increasing number of travellers returning from endemic countries who are non-adherent to malaria chemoprophylaxis [22]. Most imported cases occur in non-immune patients in whom malaria can rapidly progress to severe disease, with mortality as high as 20 to 35%, even with adequate anti-malarial treatment [23]. ARDS may develop in isolation or as part of a severe multisystem illness and often occurs within a few days of starting treatment when parasitaemia is falling [24]. The pathogenesis of ARDS in malaria is not fully understood, though it may involve parasite sequestration in the pulmonary vasculature and up-regulation of pro-inflammatory cytokines, and may persist even after parasite clearance [1]. However, parasite sequestration in the pulmonary vasculature seems to be relevant only for *P. falciparum* infection, given that in *P. vivax* malaria there is no substantial lung sequestration. Severe ARDS in patients with malaria has a poor prognosis, with mortality as high as 69 to 95% in observational ICU case series [22,25]. Severe ARDS most commonly complicates *P. falciparum* infection and is rarely a complication of *P. vivax* or *P. ovale* infection. This could be related to differences in cyto-adhesion of infected erythrocytes [26-29]. However, it should be stressed that it is difficult to definitely establish malaria as the sole etiology for severe ARDS, fully excluding bacterial co-infection. Nevertheless, no documentation of bacterial co-infection was found despite extensive microbiology specimen collection and culture at ICU admission. This could support the assumption that malaria was the most probable cause for severe ARDS in the three cases presented in this report.

Risk factors associated with the development of ARDS in malaria include advanced age, lack of specific immunity, immunocompromised status, and pregnancy. A high parasitic burden is not necessary [30]. In this case series, patients were either semi-immune or non-immune and parasitaemia was less than 4% in all patients.

There are only two prior cases reported separately on the use of ECMO for severe ARDS in patients with malaria, as well as one case on the use of extracorporeal carbon dioxide elimination [5,12,13]. In both cases of ECMO use, *P. falciparum* was identified as the causal organism and ECMO was instituted within seven days for refractory hypoxaemia, but only one patient survived [5,12]. In the case of extracorporeal carbon elimination use, *P. falciparum* was also identified as the causal organism and extracorporeal carbon elimination was instituted on the 17^th^ day, being discontinued after twelve days. The patient was weaned from mechanical ventilation and successfully discharged home [13]. In the present case series, one of the two patients that initiated ECMO after more than seven days of IMV did not survive. Patients with ARDS who have been receiving IMV with excessively high plateau pressures or high fractions of inspired oxygen for more than seven days may be less likely to benefit from ECMO [31-35]. Earlier initiation of ECMO, for these or other reasons, has been associated with better outcomes in some, but not all, observational studies [33,36-38].

Patients with severe forms of malaria are highly susceptible to bacterial infections and concomitant antibacterial therapy is recommended [2,39]. All the three patients presented in this case series received adequate antimicrobial therapy, though one patient developed nosocomial infections and ultimately died of sepsis, despite appropriate antibacterial coverage.

ECMO-related complications in the present case series included cannula migration into a hepatic vein, which was identified by transesophageal echocardiogram and corrected at the bedside. Thrombus formation in the oxygenator was ultimately the cause for oxygenator exchange in one patient. However, the rate of thrombosis-associated oxygenator failure requiring device exchange is low, even at low levels of anticoagulation [14]. Major bleeding complications, as occurred in one patient, are less frequent in the era of modern ECMO technology, given the ability to maintain circuit integrity at lower levels of anticoagulation, but they may still occur, especially in the setting of pre-existing thrombocytopaenia or coagulopathy.

In conclusion, severe ARDS can complicate *P. falciparum*, *P. vivax*, and *P. ovale* infection. In regions where the technology is available, ECMO referral should be considered for patients with malaria complicated by severe ARDS refractory to conventional treatment. Taking into account the limited experience with ECMO in severe malaria yet, its use should be performed in experienced ECMO centers. In fact, severe malaria can cause haemolysis, disseminated intravascular coagulation and low platelets, which might be exaggerated by the use of extracorporeal devices.

## Competing interests

The authors declare that they have no competing interests.

## Authors’ contributions

CA, JTC, NP, DA, RRA and DB wrote the paper. CA, PF, LS, AS, JAP and RRA were the physicians responsible for Patient 1 and Patient 2. JTC, NP, DA, MB, MLW and DB were the physicians responsible for Patient 3. RRA and DB conceived the study, its design and coordination. All authors have read and approved the final manuscript.

## References

[B1] Taylor WR Hanson J Turner GD White NJ Dondorp AM Respiratory manifestations of malaria Chest 2012 142 492 505 10.1378/chest.11-2655 22871759

[B2] Santos LC Abreu CF Xerinda SM Tavares M Lucas R Sarmento AC Severe imported malaria in an intensive care unit: a review of 59 cases Malar J 2012 11 96 10.1186/1475-2875-11-96 22458840 PMC3350412

[B3] Frickmann H Schwarz NG Holtherm HU Maassen W Vorderwulbecke F Erkens K Fischer M Morwinsky T Hagen RM Compliance with antimalarial chemoprophylaxis in German soldiers: a 6-year survey Infection 2013 41 311 320 10.1007/s15010-013-0411-5 23371855

[B4] Bruneel F Hocqueloux L Alberti C Wolff M Chevret S Bedos JP Durand R Le Bras J Regnier B Vachon F The clinical spectrum of severe imported falciparum malaria in the intensive care unit: report of 188 cases in adults Am J Respir Crit Care Med 2003 167 684 689 10.1164/rccm.200206-631OC 12411286

[B5] Losert H Schmid K Wilfing A Winkler S Staudinger T Kletzmayr J Burgmann H Experiences with severe *P. falciparum* malaria in the intensive care unit Intensive Care Med 2000 26 195 201 10.1007/s001340050045 10784308

[B6] De Nardo P Oliva A Giancola ML Ghirga P Mencarini P Bibas M Nicastri E Antinori A Corpolongo A Haemolytic anaemia after oral artemether-lumefantrine treatment in a patient affected by severe imported falciparum malaria Infection 2013 41 863 865 Epub ahead of print 10.1007/s15010-013-0451-x 23553281

[B7] Brodie D Bacchetta M Extracorporeal membrane oxygenation for ARDS in adults N Engl J Med 2011 365 1905 1914 10.1056/NEJMct1103720 22087681

[B8] Peek GJ Mugford M Tiruvoipati R Wilson A Allen E Thalanany MM Hibbert CL Truesdale A Clemens F Cooper N Firmin RK Elbourne D CESAR trial collaboration: **e**fficacy and economic assessment of conventional ventilatory support versus extracorporeal membrane oxygenation for severe adult respiratory failure (CESAR): a multicentre randomised controlled trial Lancet 2009 374 1351 1363 10.1016/S0140-6736(09)61069-2 19762075

[B9] Davies A Jones D Bailey M Beca J Bellomo R Blackwell N Forrest P Gattas D Granger E Herkes R Jackson A McGuinness S Nair P Pellegrino V Pettilä V Plunkett B Pye R Torzillo P Webb S Wilson M Ziegenfuss M Australia and New Zealand Extracorporeal Membrane Oxygenation (ANZ ECMO) Influenza Investigators Extracorporeal membrane oxygenation for 2009 Influenza A(H1N1) acute respiratory distress syndrome JAMA 2009 302 1888 1895 19822628 10.1001/jama.2009.1535

[B10] Patroniti N Zangrillo A Pappalardo F Peris A Cianchi G Braschi A Iotti GA Arcadipane A Panarello G Ranieri VM Terragni P Antonelli M Gattinoni L Oleari F Pesenti A The Italian ECMO network experience during the 2009 influenza A(H1N1) pandemic: preparation for severe respiratory emergency outbreaks Intensive Care Med 2011 37 1447 1457 10.1007/s00134-011-2301-6 21732167 PMC7080128

[B11] Noah MA Peek GJ Finney SJ Griffiths MJ Harrison DA Grieve R Sadique MZ Sekhon JS McAuley DF Firmin RK Harvey C Cordingley JJ Price S Vuylsteke A Jenkins DP Noble DW Bloomfield R Walsh TS Perkins GD Menon D Taylor BL Rowan KM Referral to an extracorporeal membrane oxygenation center and mortality among patients with severe 2009 influenza A(H1N1) JAMA 2011 306 1659 1668 10.1001/jama.2011.1471 21976615

[B12] Vandroux D Leaute B Hoarau N Ursulet L Djouhri S Braunberger E Gauzere BA High frequency oscillation ventilation and extracorporeal membrane oxygenation during pernicious malaria Med Mal Infect 2011 41 209 212 10.1016/j.medmal.2010.11.001 21194862

[B13] Neurath M Benzing A Knolle P Grundmann H Dippold W Meyer zum Buschenfelde KH Acute respiratory failure in tropical malaria during pregnancy. Successful treatment using extracorporeal CO2 elimination Dtsch Med Wochenschr 1993 118 1060 1066 10.1055/s-2008-1059426 8330507

[B14] Javidfar J Brodie D Wang D Ibrahimiye AN Yang J Zwischenberger JB Sonett J Bacchetta M Use of bicaval dual-lumen catheter for adult venovenous extracorporeal membrane oxygenation Ann Thorac Surg 2011 91 1763 1768 discussion 1769 10.1016/j.athoracsur.2011.03.002 21619973

[B15] Javidfar J Wang D Zwischenberger JB Costa J Mongero L Sonett J Bacchetta M Insertion of bicaval dual lumen extracorporeal membrane oxygenation catheter with image guidance ASAIO J 2011 57 203 205 10.1097/MAT.0b013e3182155fee 21499077

[B16] WHO Guidelines for the treatment of malaria 2010 Geneva: WHO http://whqlibdoc.who.int/publications/2010/9789241547925_eng.pdf

[B17] Ventilation with lower tidal volumes as compared with traditional tidal volumes for acute lung injury and the acute respiratory distress syndrome: the acute respiratory distress syndrome network N Engl J Med 2000 342 1301 1308 10793162 10.1056/NEJM200005043421801

[B18] White NJ Lumbar puncture in cerebral malaria Lancet 1991 338 640 641 10.1016/0140-6736(91)90654-8 1679182

[B19] Ponsford MJ Medana IM Prapansilp P Hien TT Lee SJ Dondorp AM Esiri MM Day NP White NJ Turner GD Sequestration and microvascular congestion are associated with coma in human cerebral malaria J Infect Dis 2012 205 663 671 10.1093/infdis/jir812 22207648 PMC3266137

[B20] Hanson JP Lam SW Mohanty S Alam S Pattnaik R Mahanta KC Hasan MU Charunwatthana P Mishra SK Day NP White NJ Dondorp AM Fluid resuscitation of adults with severe falciparum malaria: effects on acid–base status, renal function, and extravascular lung water Crit Care Med 2013 41 972 981 10.1097/CCM.0b013e31827466d2 23324951 PMC7616992

[B21] Wiedemann HP Wheeler AP Bernard GR Thompson BT Hayden D de Boisblanc B Connors AF Jr Hite RD Harabin AL Comparison of two fluid-management strategies in acute lung injury N Engl J Med 2006 354 2564 2575 16714767 10.1056/NEJMoa062200

[B22] Bruneel F Tubach F Corne P Megarbane B Mira JP Peytel E Camus C Schortgen F Azoulay E Cohen Y Georges H Meybeck A Hyvernat H Trouillet JL Frenoy E Nicolet L Roy C Durand R Le Bras J Wolff M Severe Imported Malaria in Adults (SIMA) Study Group: severe imported falciparum malaria: a cohort study in 400 critically ill adults PLoS One 2010 5 e13236 10.1371/journal.pone.0013236 20949045 PMC2951913

[B23] Blumberg L Lee RP Lipman J Beards S Predictors of mortality in severe malaria: a two year experience in a non-endemic area Anaesth Intensive Care 1996 24 217 223 9133196 10.1177/0310057X9602400213

[B24] Maguire GP Handojo T Pain MC Kenangalem E Price RN Tjitra E Anstey NM Lung injury in uncomplicated and severe falciparum malaria: a longitudinal study in papua, Indonesia J Infect Dis 2005 192 1966 1974 10.1086/497697 16267769 PMC2566801

[B25] Krishnan A Karnad DR Severe falciparum malaria: an important cause of multiple organ failure in Indian intensive care unit patients Crit Care Med 2003 31 2278 2284 10.1097/01.CCM.0000079603.82822.69 14501957

[B26] Price RN Tjitra E Guerra CA Yeung S White NJ Anstey NM Vivax malaria: neglected and not benign Am J Trop Med Hyg 2007 77 79 87 18165478 PMC2653940

[B27] Rojo-Marcos G Cuadros-Gonzalez J Mesa-Latorre JM Culebras-Lopez AM de Pablo-Sanchez R Acute respiratory distress syndrome in a case of Plasmodium ovale malaria Am J Trop Med Hyg 2008 79 391 393 18784231

[B28] Lee EY Maguire JH Acute pulmonary edema complicating ovale malaria Clin Infect Dis 1999 29 697 698 10.1086/598667 10530480

[B29] Anstey NM Handojo T Pain MC Kenangalem E Tjitra E Price RN Maguire GP Lung injury in vivax malaria: pathophysiological evidence for pulmonary vascular sequestration and posttreatment alveolar-capillary inflammation J Infect Dis 2007 195 589 596 10.1086/510756 17230420 PMC2532499

[B30] Jindal SK Aggarwal AN Gupta D Adult respiratory distress syndrome in the tropics Clin Chest Med 2002 23 445 455 10.1016/S0272-5231(01)00009-0 12092038

[B31] Rouby JJ Brochard L Tidal recruitment and overinflation in acute respiratory distress syndrome: yin and yang Am J Respir Crit Care Med 2007 175 104 106 10.1164/rccm.200610-1564ED 17200505

[B32] Pugin J Verghese G Widmer MC Matthay MA The alveolar space is the site of intense inflammatory and profibrotic reactions in the early phase of acute respiratory distress syndrome Crit Care Med 1999 27 304 312 10.1097/00003246-199902000-00036 10075054

[B33] Pranikoff T Hirschl RB Steimle CN Anderson HL 3rd Bartlett RH Mortality is directly related to the duration of mechanical ventilation before the initiation of extracorporeal life support for severe respiratory failure Crit Care Med 1997 25 28 32 10.1097/00003246-199701000-00008 8989172

[B34] Jackson RM Pulmonary oxygen toxicity Chest 1985 88 900 905 10.1378/chest.88.6.900 3905287

[B35] Davis WB Rennard SI Bitterman PB Crystal RG Pulmonary oxygen toxicity. Early reversible changes in human alveolar structures induced by hyperoxia N Engl J Med 1983 309 878 883 10.1056/NEJM198310133091502 6888481

[B36] Beiderlinden M Eikermann M Boes T Breitfeld C Peters J Treatment of severe acute respiratory distress syndrome: role of extracorporeal gas exchange Intensive Care Med 2006 32 1627 1631 10.1007/s00134-006-0262-y 16874497

[B37] Mols G Loop T Geiger K Farthmann E Benzing A Extracorporeal membrane oxygenation: a ten-year experience Am J Surg 2000 180 144 154 10.1016/S0002-9610(00)00432-3 11044532

[B38] Lewandowski K Rossaint R Pappert D Gerlach H Slama KJ Weidemann H Frey DJ Hoffmann O Keske U Falke KJ High survival rate in 122 ARDS patients managed according to a clinical algorithm including extracorporeal membrane oxygenation Intensive Care Med 1997 23 819 835 10.1007/s001340050418 9310799

[B39] Abreu C Santos L Poinhos R Sarmento A Acute acalculous cholecystitis in malaria: a review of seven cases from an adult cohort Infection 2013 41 821 826 Epub ahead of print 10.1007/s15010-013-0452-9 23546998

